# The relation between mental health and career-related stress among prospective graduates in higher education stage during the COVID-19 pandemic: an evidence based on network analysis

**DOI:** 10.3389/fpsyg.2024.1381846

**Published:** 2024-07-08

**Authors:** Quan Tang, Guanghui Lei, Yan Zhang, Hui Shi

**Affiliations:** ^1^Faculty of Psychology, Beijing Normal University, Beijing, China; ^2^Center for Mental Health Education, Huazhong University of Science and Technology, Wuhan, China; ^3^School of Educational Science, Huazhong University of Science and Technology, Wuhan, China; ^4^Department of Clinical Psychology, Beijing Chao-Yang Hospital, Capital Medical University, Beijing, China

**Keywords:** network analysis, mental health, depression symptoms, anxiety symptoms, career-related stress, prospective graduates

## Abstract

**Introduction:**

The outbreak of the COVID-19 pandemic has disrupted people’s routine, bringing uncertainty and stress, leading to mental health issues. This situation is particularly acute among Chinese prospective graduates in higher education stage as they cannot predict the outcomes of their studies, graduation, and career development, and therefore experience career-related stress.

**Methods:**

This study included 4041 prospective graduates in higher education stage (using handy sampling) recruited in March 2020 as participants (M_*Age*_ = 22.56, *SD* = 1.865), utilizing a Sparse Gaussian Graphical Model for regularized partial correlation network analysis of depression symptoms (by PHQ-9), anxiety symptoms (by GAD-7), and career-related stress, which aims to explore the role of career-related stress in the symptom networks of depression and anxiety among prospective graduates in their final semester.

**Results:**

The results revealed that fatigue, sad mood, and psychomotor symptoms in depression, as well as uncontrollable worry and trouble relaxing in anxiety, were central symptoms in the network. Additionally, sad mood and guilt belonging to depressive symptoms, and feeling afraid, restlessness, and irritability belonging to anxiety symptoms, served as bridge symptoms connecting symptom communities. Specifically, guilt as a depressive symptom showed a strong association with employment stress in career-related stress. There were no significant differences in network structure and global strength based on participants’ gender. However, despite no significant differences in network structure, the global strength of prospective graduates from Wuhan was significantly lower than samples from non-Hubei provinces, possibly indicative of a Typhoon Eye Effect.

**Discussion:**

The findings of this study can inspire psychological professionals in higher education institutions to provide support for mental interventions and therapies for prospective graduates, and addressing career development issues.

## Introduction

The outbreak of the COVID-19 pandemic had a huge impact on people’s lives. During this period, the countries’ economy (Goodell, 2020) and energy (Jiang et al., 2021; Szczygielski et al., 2022) were affected, companies’ performance suffered (Shen et al., 2020), and individuals’ multiple interpersonal relationships (Morelli et al., 2020; Qu et al., 2021; Chung et al., 2022; Su et al., 2022; Zhu et al., 2022; Afriat et al., 2023; Cheong et al., 2023), mental health status and well-being are also threatened (Crabtree et al., 2021; Stieger et al., 2021; Tan et al., 2021; Negri et al., 2023). Results of a systematic review by Xiong et al. (2020) indicate that during the COVID-19 pandemic, the general population in China, Spain, Italy, Iran, the United States, Turkey, Nepal, and Denmark experienced relatively high proportions of anxiety (6.33% to 50.9%), depression (14.6% to 48.3%)), post-traumatic stress disorder (7% to 53.8%), psychological distress (34.43% to 38%), and stress (8.1% to 81.9%). According to the classification of State Council Information Office of the People’s Republic of China (2020), the COVID-19 pandemic in China can be divided into five stages, which successively represent the state of sudden outbreak, spread, being controlled, interruption and normalization of the epidemic in China. As a significant public health issue, The COVID-19 pandemic forced national authorities to take various measures to control the spread of the virus during these stages, such as restricting movement (e.g., home isolation, avoiding contact with others, and shifting some work or study tasks online), strengthening medical resources, social distancing, and mandatory quarantines. Admittedly, the spread of the disease and measures to combat its transmission indeed disrupted people’s daily routines, forcing us to adapt, such as engaging in online work or study (Gutentag and Asterhan, 2022; Yang et al., 2022), online interpersonal interactions (Carballea and Rivera, 2020; Li et al., 2022; Friemel and Geber, 2023), the postponement, or even cancellation of some important plans (Toyoda et al., 2021; Zilber et al., 2023). For students, they faced threats to their study, life, health and other aspects. Research by Wu et al. (2022) identified important topics affecting students in the pandemic, including emotional reactions, influence on learning, the influence on daily life, positive responses to the COVID-19 pandemic, and China’s moves. Archibald et al. (2023) also found that physical health, emotional well-being, or ability to afford food were important stressors for students. These processes of adaptation and coping such difficulties present opportunities (Zhang et al., 2023), but also force individuals to face challenges, especially when they occur at certain stages in life.

For college students, the last semester of college is a significant period of life transition (Zou et al., 2018). In this stage, barring unforeseen circumstances, the majority of them would conclude their student identity and prepare for their career development (Chen et al., 2013). Therefore, they face career-related stress events such as completing the required credit courses within the limited time, finishing the content of the thesis or graduation project, and selecting their career path and securing relevant job positions. However, due to the COVID-19 pandemic, the Ministry of Education of the People’s Republic of China (2020) issued a notice, prohibiting graduates from returning to campus to complete their studies. This introduces uncertainty to their graduation and short-term career development, as they cannot go through their last semester of college as usual. Uncertainty refers to a state where the outcome or probability distribution of outcomes is unpredictable (Schweizer et al., 2023). For these prospective university graduates, the outward manifestation highlighted in the uncertainty brought by the COVID-19 pandemic is career-related stress and impact. Previous research has provided ample evidence for the relationship between uncertainty and mental health issues (Junca-Silva and Silva, 2022; Massazza et al., 2023; Schweizer et al., 2023). However, after we searched the web of science using “network analysis”, “mental health” and “career related stress” as keywords, we found that there were no researches exploring the position of these career-related stress and impacts in the symptom networks of depression and anxiety. Therefore, this study used regularized partial correlation network analysis to explore the relationship between career-related stress and symptoms of depression and anxiety among prospective graduates who cannot return to campus due to COVID-19 in their last semester of college life.

### Career-related stress and mental health

College students are recognized as one of the groups significantly affected by stress, and they will face numerous potential sources of stress (Darling et al., 2007), such as economic, family, interpersonal relationships, academic, and career development. Among these, career-related concerns are one of the common topics for psychological counseling among college students (Benton et al., 2003). Career-related stress refers to the negative emotions individuals experience when encountering adverse experiences in setting and pursuing career goals (Creed et al., 2016). Undeniably, the outbreak of the COVID-19 pandemic has indeed impacted people’s career development, career commitment, and career regrets (Huo, 2021). For prospective graduates, the outbreak of the pandemic has disrupted their usual routines in the field of career development, forcing them to endure career-related stress (Peng Y. et al., 2023). In particular, a study by Peng Y. et al. (2023) found that about 13.2% of participants felt depressed, and 53.3% of them believed that the current employment situation is grim.

Stress is one of the crucial predictive factors for people’s mental health (Guntzviller et al., 2020). Zhang et al. (2021) noted in their study that the COVID-19 pandemic has brought a considerable level of stress to Chinese university students, accompanied by emotional and somatic responses, and the level of stress also varied at different time periods. Zhou et al. (2022) highlighted in their work that uncertainty is a crucial factor in the career stress of prospective university graduates, which is because COVID-19 can impact the economy and employment situation, hindering their transition from school to the workplace and causing difficulties in determining their employment prospects and career opportunities. During the COVID-19 pandemic, numerous studies have discussed the association between college students’ career stress and their mental health. For instance, research by Okado et al. (2023) found that participants reported increased concerns about academic and employment issues, and concurrently, they also reported a significant elevation in depression, anxiety, perceived stress, and somatic symptoms. Additionally, the validated chain mediation model in Zhou et al. (2022) suggests that tolerance for uncertainty can positively predict fear of COVID-19, and this fear, in turn, can positively predict depression, subsequently predicting future career anxiety. Lv et al. (2023) also found in their study that the impact of the COVID-19 pandemic on career planning positively predicted anxiety symptoms.

### Psychometric network

In recent years, network analysis has been widely used in the field of psychopathology research (Contreras et al., 2019). In traditional views, researchers in psychopathology considered mental disorders as combinations of symptoms or latent categories or dimensions constituted by symptoms (Borsboom, 2008). However, Borsboom (2008) pointed out that existing mental disorders might be a causal system constituted by symptoms. In other words, the correlations among symptoms within a disorder are not incidental and are greater than the correlations between symptoms of different disorders. Network analysis is a powerful tool used to reveal this system because it can demonstrate the interactions among symptoms (Beard et al., 2016). Networks typically consist of two essential components: nodes and edges. Nodes, visualized as circles within the network, represent variables existing in the network (e.g., symptoms of a particular mental disorder); edges, visualized as lines with varying thickness or color connecting nodes, represent relationships between variables in the network (e.g., correlations or partial correlations) (Borsboom and Cramer, 2013). Through network analysis, researchers can identify central and bridge symptoms of specific mental disorders (Bai et al., 2022). Central symptoms are those with strong connections to surrounding nodes, while bridge symptoms are those positioned between multiple symptoms of mental disorders, with a higher risk of extending one disorder to others (Cramer et al., 2010b). These symptoms play a crucial role in the treatment and prevention of comorbidities in mental disorders.

Due to COVID-19 being a global significant public health event with crucial implications for research in psychopathology and mental health, previous studies have utilized network analysis to discuss the network structures of various mental disorders during the pandemic. A study by Fico et al. (2023) on a Spanish population, anxiety and depression exhibited higher centrality in COVID-19 patients, with anxiety having the highest bridge expected influence in the network. Moreover, there were no statistically significant differences in such network results between participants with or without any history of mental disorders and those who had or hadn’t been hospitalized. Peng P. et al. (2023) found in their study on Chinese COVID-19 survivors that the most prominent symptoms included uncontrollable and excessive worry, psychomotor symptoms, intrusion, and daytime dysfunction. Additionally, daytime dysfunction and fatigue were identified as bridge symptoms in the mental disorder network and exhibited a strong association with quality of life. It is worth mentioning that Ventura-León et al. (2022)’s study found that depressive symptoms were considered the central and bridging elements in the network, interrelated with some symptoms of stress and anxiety. Specifically, sad mood in depressive symptoms, nervousness in anxiety symptoms, and the item “you felt difficulties were piling up so high that you could not overcome them” in perceived stress were each identified as central nodes, while sad mood and concentration in depressive symptoms were bridge symptoms connecting different symptom communities. However, there were no studies discussing the specific associations between career-related stress and the symptoms of depression and anxiety, as well as its position in the symptom network.

### Current study

The aim of this study is to explore the relationship between career-related stress and mental health among prospective graduates in Chinese higher education during the COVID-19 pandemic. To this end, we utilized cross-sectional data collected from a sample of prospective graduates in higher education in March 2020 (typically the early phase of the spring semester in 2020). A Sparse Gaussian Graphical model was used to conduct regularized partial correlation network analysis of depressive symptoms, anxiety symptoms, and career-related stress, with the goal of identifying central and bridge symptoms within the network. Additionally, we sought to explore the prominence of career-related stress highlighted by the pandemic within the symptom network, as well as its direct and indirect connections to depressive and anxiety symptoms. The exploration of psychometric network provides support for identifying key factors related to college students’ mental health and protecting their well-being in response to similar public health events.

## Methods

### Participants and data collection

The sample for this study came from an online survey conducted on prospective graduates in Chinese higher education stage in the year 2020. The data collection period was from March 11 to March 20, 2020. At this point, all higher education institutions in China had not officially resumed classes, and students were not allowed to return to campus, because of the notification issued by the Ministry of Education of the People’s Republic of China (2020). All questionnaires were distributed through the Wenjuanxing^1^ on various online social media platforms, including WeChat, QQ, and Sina microblog. Participants answered the questionnaire on their mobile phones following the instructions provided, and each participant could only fill out the questionnaire once. All responses were submitted anonymously. The sample consisted of 4041 prospective college graduates in China (*M*_Age_ = 22.56, *SD* = 1.865, ranging from 18 to 52 and containing 235 people who did not report valid age information) with 2109 of them being female. This online survey received ethical approval from the Ethics Committee of Huazhong University of Science and Technology.

### Measurements

#### Depression symptoms

We used the Patient Health Questionnaire-9 items (PHQ-9) developed by Kroenke et al. (2001) as the assessment tool for depression symptoms in the participants (current Cronbach’s α = 0.935, McDonald’s ω = 0.939). PHQ-9 is a single-dimensional 4-point scale (ranging from 0 = *not at all* to 3 = *almost every day*), consisting of 9 items (e.g., “Little interest or pleasure in doing things.”). Participants were asked to report the frequency of symptoms described in the items occurring in the past 2 weeks. A higher score on this scale indicates a higher frequency of depressive symptoms exhibited by participants in the recent two weeks.

#### Anxiety symptoms

The 7-item general anxiety disorder scale (GAD-7) developed by Spitzer et al. (2006) was used to measure the symptoms of anxiety in this study (current Cronbach’s α = 0.955, McDonald’s ω = 0.956). GAD-7 is a single-dimensional scale consisting of 7 items (e.g., “Feeling nervous, anxious or on edge.”). Participants are required to report the frequency of symptoms described in each item on a 4-point scale (ranging from 0 = *not at all* to 3 = *almost every day*) occurring in the past 2 weeks. A higher score on this scale indicates a higher frequency of general anxiety symptoms exhibited by participants in the recent two weeks.

#### Career-related stress

In this study, participants’ career-related stress and the impact caused by the pandemic were assessed using a self-designed questionnaire with 8 items, which was also used in the research by Zhong et al. (2024). The questionnaire, on a 5-point scale, is divided into two dimensions: career-related stress (CRS, current Cronbach’s α = 0.805, McDonald’s ω = 0.805) and career-related impact (CRI, current Cronbach’s α = 0.636, McDonald’s ω = 0.679). CRS consists of 5 items (e.g., “Do you feel pressure in employment?”) involving learning, thesis, employment, life, and entrance exams; CRI includes 3 items (e.g., “Has the epidemic had an impact on your job seeking?”) related to thesis, job seeking, and online recruitment. Participants evaluated the stress and impact they experienced during the delayed school opening on a scale of 1 = *no stress* to 5 = *serious stress* and 1 = *no impact* to 5 = *serious impact*, with higher scores indicating greater stress and more severe impact.

#### Demographical information

We collected participants’ gender (1 = male, 2 = female), age, health status (1 = Health; 2 = Suspected COVID-19 Symptoms; 3 = Confirmed COVID-19 Infection; 4 = Recovery from COVID-19; 5 = Cold Symptom), place of residence (1 = Wuhan City; 2 = Other Parts of Hubei Province; 3 = Other Provinces), and school location (1 = Wuhan City; 2 = Other Parts of Hubei Province; 3 = Other Provinces) as their demographic information.

### Statistical analysis

We used IBM SPSS Statistics 26 for data management, descriptive statistical analysis, and correlation analysis. For the regularization partial correlation network analysis and network comparison test, we employed R 4.3.2 and R packages including *qgraph* (Epskamp et al., 2012), *bootnet* (Epskamp et al., 2018), *networktools* (Jones et al., 2021), *mgm* (Haslbeck and Waldorp, 2020), and *NetworkComparisonTest* (Van Borkulo et al., 2022). GAD-7, PHQ-9, and the career-related stress questionnaire with a total of 24 items were included as nodes in the analysis. The node naming followed the studies of Garabiles et al. (2019) and Bai et al. (2022). According to the suggestion by Epskamp and Fried (2018), Sparse Graphical Gaussian Models were used for the network analysis in this study. And due to the ordinal nature of item scores, Spearman partial correlation was used, regularized by the least absolute shrinkage and selection operator (lasso; Tibshirani, 1996) to prevent spurious correlations and achieve model sparsity. Extended Bayesian Information Criterion (EBIC; Chen and Chen, 2008) was utilized for model selection, with the hyperparameter γ set to 0.5 following the recommendation of Foygel and Drton (2010).

Besides, we also computed centrality for each node, including strength, betweenness, closeness, and expected influence. Larger values of these indicators signify greater importance of the corresponding nodes. Additionally, we calculated the bridge expected influence for each node to represent the risk of spreading from one community to others (Jones et al., 2021). Nodes with Bridge expected influence values in the top 20% were selected as bridge symptoms (Jones et al., 2021). Predictability was also computed to indicate the degree of association between each node and its neighboring nodes.

The stability and accuracy of the network model will be evaluated through non-parametric bootstrap and case-dropping bootstrap (both with 5000 bootstrap samples). Using non-parametric bootstrap, we calculated the 95% confidence interval for each edge to ensure that they are significantly different from others. Bootstrap difference tests for expected influence of each node were also conducted to ensure the uniqueness of nodes. The stability of centrality was assessed through case-dropping bootstrap, calculating the correlation stability coefficient (CS) for each centrality coefficient (including strength, betweenness, closeness, expected influence, bridge strength, and bridge expected influence). Following the recommendation of Epskamp and Fried (2018), achieving a CS of 0.5 is considered ideal with at least 0.25 being acceptable, ensuring at least a 0.7 correlation with the original coefficients at a 95% probability.

Considering the influence of gender and the location of the epidemic, we conducted significance tests (permutations = 1,000) to compare networks composed of samples with different genders and different places of residence. Since we have no a priori hypotheses, specific edge difference tests will not be included in this study.

## Results

### Preliminary analysis

The demographic characteristics of the sample are shown in Table 1 and Supplementary Table 1. We calculated the mean, standard deviation, and correlations between depression, anxiety symptoms, and career-related stress, as presented in Table 2.

**TABLE 1:** Demographic information (*N* = 4041).

	*n*	%
**Gender**		
Male	1932	47.81
Female	2019	49.96
**Health Status**		
Health	4019	99.46
Suspected COVID-19 Symptoms	1	0.02
Confirmed COVID-19 Infection	1	0.02
Recovery from COVID-19	2	0.05
Cold Symptom	18	0.45
**Place of Residence**		
Wuhan City	108	2.67
Other Parts of Hubei Province	353	8.74
Other Provinces	3580	88.59
**School Location**		
Wuhan City	1130	27.96
Other Parts of Hubei Province	152	3.76
Other Provinces	2759	68.28

**TABLE 2:** Descriptive statistics and correlation matrices.

	GAD	PHQ	CRS	CRI
GAD	–			
PHQ	0.822	–		
CRS	0.363	0.361	–	
CRI	0.425	0.425	0.429	–
*M*	0.924	0.811	3.068	3.179
*SD*	0.879	0.769	0.955	0.941
Range	0∼3	0∼3	1∼5	1∼5

GAD, 7-Item General Anxiety Disorder Scale; PHQ, Patient Health Questionnaire-9 items; CRS, career-related stress; CRI, career-related impact. All correlation coefficients are significant at the α = 0.001.

### Network structure

**FIGURE 1:**
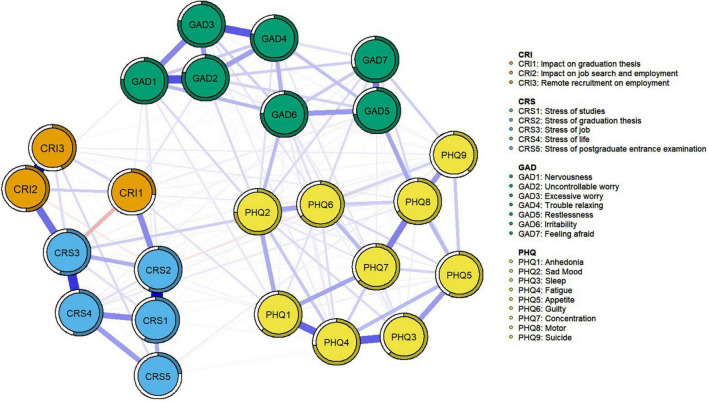
Psychometric Network. The ring around a node represents predictability; The thickness of the line represents the strength of the relationship; The color of the line represents the positive and negative of the relationship, with red being negative and blue being positive.

The regularized partial correlation network comprises 24 nodes, with 160 non-zero edges, accounting for 57.97% of the total 276 edges. The average weight of edges is .039. The ten strongest edges are mainly distributed within various symptom communities, including 3 for depression symptoms, 3 for anxiety symptoms, 3 for career-related stress, and 1 for career-related impact. The edges with the highest weights are CRS1 (stress of studies) and CRS2 (stress of graduation thesis), followed by CRI2 (impact on job search and employment) and CRI3 (remote recruitment on employment). Subsequently, in decreasing order, there are CRS3 (stress of job) and CRS4 (stress of life); GAD1 (nervousness) and GAD2 (uncontrollable worry); GAD3 (excessive worry) and GAD4 (trouble relaxing); PHQ3 (sleep) and PHQ4 (fatigue); PHQ1 (anhedonia) and PHQ4 (fatigue); CRS3 (stress of job) and CRI2 (impact on job search and employment); GAD5 (restlessness) and GAD7 (feeling afraid); PHQ7 (concentration) and PHQ8 (motor). The regularized partial correlation coefficients are presented in Table 3, and the network structure is visualized in Figure 1. Predictability ranges from 79.1% to 23.4%, with an average predictability of 60.8%, indicating that, on average, 60.8% of the variance in each node can be explained by its surrounding nodes. The highest predictability is observed in anxiety symptoms, in descending order: GAD2 (uncontrollable worry), GAD4 (trouble relaxing), and GAD3 (excessive worry); the lowest predictability occurs in career-related stress and impact, as well as depression symptoms, in ascending order: CRS5 (stress of postgraduate entrance examination), CRI1 (Impact on graduation thesis), and PHQ9 (suicidal ideation). The predictability of other nodes is detailed in Table 3.

**TABLE 3:** Regularized partial correlation matrix, centrality coefficients, and bridge centrality coefficients.

	GAD 1	GAD 2	GAD 3	GAD 4	GAD 5	GAD 6	GAD 7	PHQ 1	PHQ 2	PHQ 3	PHQ 4	PHQ 5	PHQ 6	PHQ 7	PHQ 8	PHQ 9	CRS 1	CRS 2	CRS 3	CRS 4	CRS 5	CRI 1	CRI 2	CRI 3
GAD1	0.000	0.335	0.230	0.102	0.000	0.137	0.001	0.042	0.073	0.000	0.001	0.000	0.000	0.000	0.000	−0.019	0.000	0.042	0.000	0.000	−0.036	0.033	0.000	0.000
GAD2	0.335	0.000	0.197	0.181	0.085	0.040	0.096	0.000	0.070	0.004	0.000	0.001	0.015	0.002	0.000	0.000	0.000	0.001	0.000	0.000	0.000	0.052	0.000	0.000
GAD3	0.230	0.197	0.000	0.319	0.000	0.091	0.060	0.000	0.012	0.008	0.036	0.000	0.049	0.000	0.000	−0.019	0.012	0.000	0.000	0.000	0.000	0.000	0.036	0.000
GAD4	0.102	0.181	0.319	0.000	0.119	0.147	0.051	0.000	0.058	0.045	0.026	0.000	0.000	0.006	0.000	0.000	0.014	0.006	0.000	0.000	0.000	0.006	0.000	0.000
GAD5	0.000	0.085	0.000	0.119	0.000	0.196	0.260	0.000	0.005	0.009	0.000	0.055	0.000	0.031	0.169	0.017	0.015	0.000	−0.023	0.017	0.000	0.034	0.000	0.000
GAD6	0.137	0.040	0.091	0.147	0.196	0.000	0.100	0.021	0.129	0.046	0.039	0.000	0.061	0.032	0.000	0.000	0.004	0.003	0.000	0.000	−0.009	0.003	0.000	0.000
GAD7	0.001	0.096	0.060	0.051	0.260	0.100	0.000	0.000	0.071	0.014	0.003	0.000	0.069	0.000	0.072	0.095	0.000	0.000	0.000	0.024	0.000	0.010	0.007	0.008
PHQ1	0.042	0.000	0.000	0.000	0.000	0.021	0.000	0.000	0.162	0.012	0.298	0.028	0.058	0.176	0.000	0.000	0.000	0.020	−0.020	0.000	0.000	0.057	0.000	0.000
PHQ2	0.073	0.070	0.012	0.058	0.005	0.129	0.071	0.162	0.000	0.068	0.105	0.029	0.169	0.049	0.062	0.023	0.000	0.000	0.000	0.010	0.000	0.000	0.014	0.000
PHQ3	0.000	0.004	0.008	0.045	0.009	0.046	0.014	0.012	0.068	0.000	0.309	0.192	0.030	0.046	0.029	0.000	0.000	0.000	0.000	0.000	−0.010	0.000	0.000	0.000
PHQ4	0.001	0.000	0.036	0.026	0.000	0.039	0.003	0.298	0.105	0.309	0.000	0.135	0.064	0.098	0.014	0.000	0.013	0.000	0.000	0.000	−0.002	0.000	0.000	0.000
PHQ5	0.000	0.001	0.000	0.000	0.055	0.000	0.000	0.028	0.029	0.192	0.135	0.000	0.074	0.080	0.135	0.104	0.000	0.000	−0.012	0.017	0.000	0.000	0.000	0.015
PHQ6	0.000	0.015	0.049	0.000	0.000	0.061	0.069	0.058	0.169	0.030	0.064	0.074	0.000	0.130	0.096	0.082	0.007	0.000	0.093	0.013	0.000	0.000	0.035	0.000
PHQ7	0.000	0.002	0.000	0.006	0.031	0.032	0.000	0.176	0.049	0.046	0.098	0.080	0.130	0.000	0.244	0.000	0.003	0.014	−0.006	0.000	0.000	0.016	0.000	0.000
PHQ8	0.000	0.000	0.000	0.000	0.169	0.000	0.072	0.000	0.062	0.029	0.014	0.135	0.096	0.244	0.000	0.196	0.000	0.000	−0.017	0.038	0.010	0.000	0.000	0.037
PHQ9	−0.019	0.000	−0.019	0.000	0.017	0.000	0.095	0.000	0.023	0.000	0.000	0.104	0.082	0.000	0.196	0.000	0.000	−0.051	−0.020	0.025	0.032	0.018	0.000	0.034
CRS1	0.000	0.000	0.012	0.014	0.015	0.004	0.000	0.000	0.000	0.000	0.013	0.000	0.007	0.003	0.000	0.000	0.000	0.496	0.058	0.227	0.157	0.000	−0.008	−0.010
CRS2	0.042	0.001	0.000	0.006	0.000	0.003	0.000	0.020	0.000	0.000	0.000	0.000	0.000	0.014	0.000	−0.051	0.496	0.000	0.155	0.058	0.000	0.223	−0.041	−0.009
CRS3	0.000	0.000	0.000	0.000	−0.023	0.000	0.000	−0.020	0.000	0.000	0.000	−0.012	0.093	−0.006	−0.017	−0.020	0.058	0.155	0.000	0.389	0.015	−0.139	0.282	0.066
CRS4	0.000	0.000	0.000	0.000	0.017	0.000	0.024	0.000	0.010	0.000	0.000	0.017	0.013	0.000	0.038	0.025	0.227	0.058	0.389	0.000	0.190	0.000	0.000	0.032
CRS5	−0.036	0.000	0.000	0.000	0.000	−0.009	0.000	0.000	0.000	−0.010	−0.002	0.000	0.000	0.000	0.010	0.032	0.157	0.000	0.015	0.190	0.000	0.000	0.000	0.070
CRI1	0.033	0.052	0.000	0.006	0.034	0.003	0.010	0.057	0.000	0.000	0.000	0.000	0.000	0.016	0.000	0.018	0.000	0.223	−0.139	0.000	0.000	0.000	0.096	0.083
CRI2	0.000	0.000	0.036	0.000	0.000	0.000	0.007	0.000	0.014	0.000	0.000	0.000	0.035	0.000	0.000	0.000	−0.008	−0.041	0.282	0.000	0.000	0.096	0.000	0.472
CRI3	0.000	0.000	0.000	0.000	0.000	0.000	0.008	0.000	0.000	0.000	0.000	0.015	0.000	0.000	0.037	0.034	−0.010	−0.009	0.066	0.032	0.070	0.083	0.472	0.000
Predictability	0.761	0.791	0.778	0.781	0.721	0.756	0.692	0.625	0.746	0.611	0.725	0.572	0.660	0.637	0.672	0.384	0.582	0.569	0.551	0.542	0.234	0.268	0.495	0.431
Strength	1.050	1.080	1.068	1.081	1.035	1.058	0.393	0.894	1.11	0.820	1.143	0.876	1.045	0.933	1.118	0.736	1.052	1.121	1.295	1.039	0.531	0.772	0.992	0.836
Closeness (×10^–2^)	0.214	0.221	0.205	0.213	0.236	0.240	0.224	0.250	0.273	0.200	0.217	0.218	0.288	0.263	0.265	0.237	0.205	0.217	0.233	0.213	0.181	0.223	0.205	0.188
Betweenness	14	20	10	22	56	30	2	80	82	0	36	10	124	18	72	16	16	40	130	36	0	38	38	0
Expected influence	0.940	1.080	1.031	1.081	0.989	1.039	0.939	0.854	1.110	0.801	1.138	0.853	1.045	0.921	1.084	0.518	0.989	0.918	0.822	1.039	0.416	0.494	0.893	0.798
Bridge strength	0.246	0.146	0.172	0.163	0.375	0.347	0.371	0.160	0.443	0.135	0.121	0.099	0.342	0.110	0.342	0.331	0.086	0.412	0.678	0.176	0.169	0.593	0.423	0.281
Bridge closeness	0.040	0.041	0.038	0.040	0.045	0.046	0.044	0.045	0.056	0.036	0.039	0.040	0.059	0.049	0.049	0.043	0.040	0.042	0.046	0.042	0.035	0.047	0.044	0.040
Bridge betweenness	4	9	3	8	25	14	1	31	38	0	12	2	72	6	34	0	11	23	74	22	0	20	19	0
Bridge expected influence (1-step)	0.136	0.146	0.134	0.163	0.330	0.329	0.371	0.120	0.443	0.116	0.116	0.076	0.342	0.098	0.308	0.112	0.050	0.209	0.205	0.176	0.055	0.314	0.325	0.243

**FIGURE 2:**
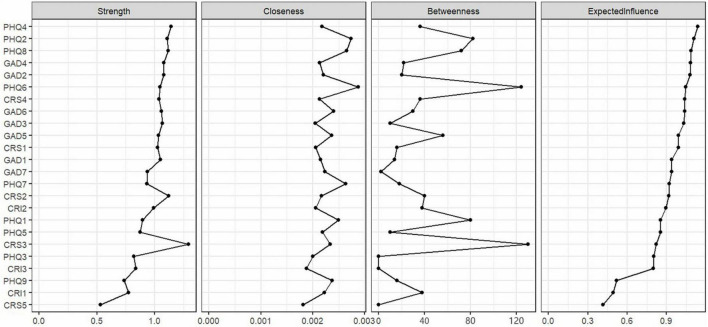
Centrality of each node.

**FIGURE 3:**
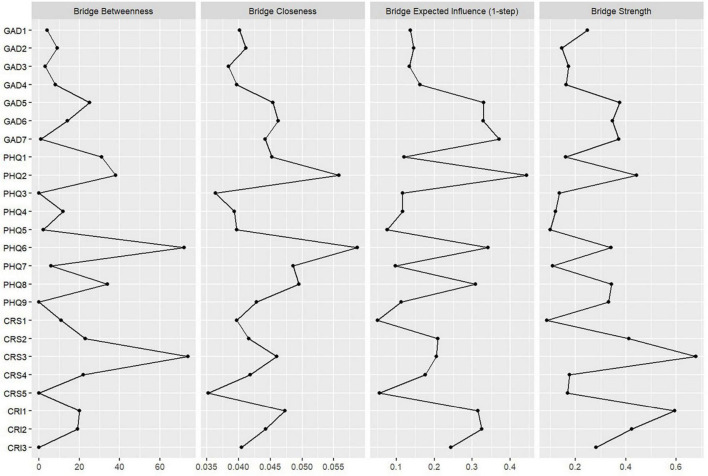
Bridge centrality of each node.

The values of strength, betweenness, closeness, and expected influence for each node are presented in Table 3 and Figure 2. Among them, PHQ4 (fatigue) has the highest expected influence, followed by PHQ2 (sad mood), PHQ8 (motor), GAD4 (trouble relaxing), and GAD2 (uncontrollable worry). This indicates that these symptoms have the greatest impact on variability in the network structure. Additionally, CRS3 (stress of job) stands out with the highest strength, indicating its closest association with surrounding symptoms. Node PHQ6 (guilty) has the highest closeness, signifying its closest proximity to other surrounding symptom nodes. Both CRS3 (stress of job) and PHQ6 (guilty) hold the highest and second-highest betweenness, respectively, indicating that the shortest paths between nodes pass through them the most. Furthermore, PHQ2 (sad mood), GAD7 (feeling afraid), PHQ6 (guilty), GAD5 (restlessness), and GAD6 (irritability) consecutively exhibit the highest bridge expected influence (see Figure 3 and Table 3). Moreover, CRS3 (stress of job) has the highest bridge strength and bridge betweenness, while PHQ6 (guilty) boasts the highest bridge closeness among all nodes and ranks second in bridge betweenness.

Edges across symptom groups are distributed in the depression-anxiety symptoms group (36 edges), career related stress-depression symptoms group (29 edges), and career related stress-anxiety symptoms group (22 edges). Among them, the top 10 edges with the highest weights are: GAD5 (restlessness)-PHQ8 (motor); GAD6 (irritability)-PHQ2 (sad mood); GAD7 (feeling afraid)-PHQ9 (suicide); PHQ6 (guilty)-CRS3 (stress of job); GAD1 (nervousness)-PHQ2 (sad mood); GAD7 (feeling afraid)-PHQ8 (motor); GAD7 (Feeling afraid)-PHQ2 (Sad Mood); GAD2 (uncontrollable worry)-PHQ2 (sad mood); GAD7 (feeling afraid)-PHQ6 (guilty); GAD6 (irritability)-PHQ6 (guilty).

### Network stability

The non-parametric bootstrap results for the edges are all significant, indicating significant differences between edges (see Supplementary Figure 1 and Supplementary Figure 2 in Supplementary materials). The test results for expected influence also significantly indicate differences between nodes (see Supplementary Figure 3). The CS for Strength, betweenness, closeness, expected influence, bridge strength, and bridge expected influence are 0.75, 0.361, 0.594, 0.75, 0.75, and 0.75, respectively. Case-dropping bootstrap for Strength, betweenness, closeness, expected influence can be seen in Supplementary Figure 4, and case-dropping bootstrap for bridge strength and bridge expected influence can be seen in Supplementary Figure 5. These results meet the empirical standards, indicating that removing 75%, 36.1%, 59.4%, 75%, 75%, and 75% of the participants would lead to correlations between the corresponding centrality coefficients and the original coefficients lower than 0.7.

### Network comparison

We compared participants of different genders (see Supplementary Figure 6) and different locations (see Supplementary Figure 7) in the symptom network through bootstrap. The results indicate no significant differences between participants of different genders in network structure (M = 0.085, *p* = 0.612) and global strength (male = 11.874, female = 11.417, S = 0.458, *p* = 0.196). However, we found a significant difference in global strength between participants living in Wuhan and those in other provinces (living in Wuhan = 10.380, living in other province = 12.200, S = 1.819, *p* = 0.039), although there is no significant difference in network structure between the two groups (M = 0.201, *p* = 0.742). At the node level, significant differences in strength were found in GAD5 (Restlessness; C = −0.273, *p* = 0.018), PHQ9 (Suicide; C = −0.584, *p* = 0.005), CRS1 (stress of studies; C = −0.324, *p* = 0.039), with participants living in Wuhan having significantly lower node strength than those living in other provinces. Additionally, significant differences in expected influence were found in PHQ9 (Suicide; C = −0.266, *p* = 0.018), with participants living in Wuhan having significantly lower expected influence on these nodes than those living in other provinces. Meanwhile, there were no significant differences in network structure (M = 0.204, *p* = 0.752) and global strength (living in Hubei = 11.126, S = 0.746, *p* = 0.158) between participants living in Wuhan and those living in other cities in Hubei province, and no significant differences in network structure (M = 0.179, *p* = 0.092) and global strength (S = 1.074, *p* = 0.259) between participants living in other cities in Hubei province and those in other provinces.

## Discussion

In this study, we explored the network of depression and anxiety symptoms among Chinese prospective graduates in higher education stage, along with the role of career-related stress in it. Consistent with previous research, the strongest edges were found primarily within the communities of mental disorders instead of connecting different mental disorders (Beard et al., 2016; Garabiles et al., 2019; Bai et al., 2022). In our results, strongly associated pairs such as GAD1 (nervousness) and GAD2 (uncontrollable worry); GAD3 (excessive worry) and GAD4 (trouble relaxing); PHQ3 (sleep) and PHQ4 (fatigue); PHQ1 (anhedonia) and PHQ4 (fatigue); GAD5 (restlessness) and GAD7 (feeling afraid); PHQ7 (concentration) and PHQ8 (motor) were also found, consistent with previous studies (Cramer et al., 2010a; Beard et al., 2016; Schuler et al., 2018; Garabiles et al., 2019; Bai et al., 2022).

In addition to depression and anxiety symptoms, we also identified connections among sources of career-related stress. Specifically, variables representing the end of student life [CRS1 (stress of studies) and CRS2 (stress of graduation thesis)] and entering the workforce [CRI2 (impact on job search and employment) and CRI3 (remote recruitment on employment); CRS3 (stress of job) and CRS4 (stress of life); CRS3 (stress of job) and CRI2 (impact on job search and employment)] were found to have strong associations within their respective communities. This aligns with previous research and the consensus that the school-to-work transition involves both the end of education and the beginning of employment (Ng and Feldman, 2007; Blokker et al., 2023). During this transition, changes in role identity, self-efficacy, and motivation occur (Pinquart et al., 2003; Ng and Feldman, 2007; Symonds et al., 2019), distinguishing between the phases of schooling and working. Importantly, we also identified that the edge with the strongest association between mental health symptoms and career-related stress is PHQ6 (guilty)-CRS3 (stress of job). This aligns with results from variable relationship studies indicating a connection between depressive symptoms and employment status (Kretzler et al., 2021; Mojtahedi et al., 2021; Levy, 2022), especially guilty and employment (Leana and Feldman, 1995; Extremera and Rey, 2014). Considering their high bridge centrality, our results may explain the connection between employment issues and depressive symptoms, suggesting that individuals experiencing career-related stress are more likely to feel guilty and, consequently, develop depression.

Our results indicate that PHQ4 (fatigue), PHQ2 (sad mood), PHQ8 (motor), GAD4 (trouble relaxing), and GAD2 (uncontrollable worry) have the highest expected influence, suggesting their strong impact on the entire symptom network. The analysis of central symptoms for depression and anxiety aligns with Bai et al. (2022), where Fatigue is a central symptom in the depression symptom community, and Trouble relaxing is central in the anxiety symptom community. We note that central symptoms in both symptom communities involve physiological depletion and lack of energy, which may be related to the prevalence of somatic symptoms in the Asian population (Chentsova-Dutton et al., 2014). Additionally, factor analysis on Chinese employees’ major depressive symptoms Mordeno et al. (2018) found that, unlike populations in other cultures where depressive symptoms are a single dimension, Chinese people’s depressive symptoms can be divided into emotional symptoms and somatic symptoms. In terms of career-related stress, CRS4 (stress of life) has the highest expected influence. This may be because stress assessments related to specific career-related tasks can be incorporated into assessments of general life event stress (Wu et al., 2020).

In this study, the identified bridge symptoms in our network are PHQ2 (sad mood), GAD7 (feeling afraid), PHQ6 (guilty), GAD5 (restlessness), and GAD6 (irritability). While sad mood as a bridge symptom in depression symptoms is supported by previous research (Garabiles et al., 2019; Jones et al., 2021), the identification of guilty as a bridge symptom is novel. Considering the association between guilt and employment stress discussed earlier, we propose that guilt and self-blame may be crucial symptoms linking career-related stress and depression symptoms. Feeling afraid, Restlessness, and Irritability, identified as bridge symptoms, are consistent with prior research (Garabiles et al., 2019; Bai et al., 2021, 2022). We note a strong positive association between Sad Mood and Irritability, both being mood-related issues, a relationship documented in previous studies in adult and child populations (Fava et al., 2010; Parneix et al., 2014; Vance and Winther, 2021). Moreover, Fava et al. (2010) pointed out in their work that irritability is particularly common among respondents and students aged 18-44. In addition, we found a significant negative correlation between restlessness and employment stress and a significant positive correlation between restlessness and the impact of the pandemic on graduation theses. Restlessness is considered to be associated with attention problems (Aitken and Andrade, 2021) suggesting that prospective graduates experiencing restlessness may struggle to concentrate on stress of employment, but the actual constant effect of their graduation theses would not be disturbed and make them anxious. Furthermore, guilty, in addition to its association with employment stress, is linked to the pandemic’s impact on employment, consistent with our explanation above.

We also examined the edge weights and global strength of depression-anxiety-career-related stress in different groups. Consistent with previous studies, our network did not reveal significant gender differences in either edge weights or global strength (Bai et al., 2021, 2022; Cheung et al., 2021). However, we observed differences in global strength among samples from different locations, even though their network structures did not change significantly. The network global strength of prospective graduates living in Wuhan was lower than that of prospective graduates from other cities in Hubei province, especially on the nodes GAD5 (restlessness), PHQ9 (suicide), and CRS1 (stress of studies). We believe this may be related to the typhoon eye effect of the psychological impact of COVID-19 found in previous studies (Wang et al., 2020; Zhang et al., 2020). As people at the center of a risk event have lower awareness of the risk event than those in surrounding areas, the non-common variation between symptoms is greater, and the links between nodes in the network are weaker.

## Limitations

Although this study provides evidence for a relationship between career-related stress and symptoms of depression and anxiety, there are still some limitations should be noted. First of all, this study used cross-sectional data and couldn’t make causal inferences. To further explore the role of career-related stress in the network of depressive and anxiety symptoms, future researchers can use longitudinal data for analysis to enhance the understanding of the relationship between stress and mental health. Second, we used self-reported questionnaire data from participants. Future researchers may consider using behavioral experiments or psychophysiological approaches to further discuss the relationship between stress and symptoms of psychiatric disorders. Finally, our sample is limited only to prospective graduates of higher education in China. Future researchers can consider using samples from other social and cultural backgrounds or conducting cross-cultural network comparisons, which would be helpful to understand and distinguish which characteristics of students have a stronger relationship between career-related stress and psychological disorder symptoms, and provide more effective intervention or treatment for them.

## Conclusion

This study delves into the relationship between career-related stress and mental health among prospective graduates in Chinese higher education during the COVID-19 pandemic. Utilizing network analysis, we revealed the important role of career-related stress within the networks of depressive and anxiety symptoms and identified central and bridge symptoms. Fatigue, sad mood, and psychomotor symptoms were identified as central symptoms of depression, as well as trouble in resting and uncontrollable worry were central symptoms of anxiety. Furthermore, sad mood and guilt served as bridge symptoms for depression, as well as feeling afraid, restlessness, and irritability served as bridge symptoms for anxiety. Notably, career-related stress, especially in terms of employment, showed a strong association with guilt. The findings also indicated no significant differences in network structure and global strength between male and female participants. However, the global strength was significantly lower among prospective graduates from Wuhan compared to those from non-Hubei provinces, potentially indicating a “Typhoon Eye Effect” in the pandemic’s epicenter.

The results of this study underscore the need for psychological professionals in higher education institutions to focus on the above symptoms when providing interventions and treatments for graduating students. Additionally, given the “Typhoon Eye Effect,” mental health support resources should be allocated appropriately across different regions, with particular attention to prospective graduates living in provinces and cities outside Wuhan, as their mental health were more strongly linked to risks factors.

## Data availability statement

The raw data supporting the conclusions of this article will be made available by the authors, without undue reservation.

## Ethics statement

The studies involving humans were approved by Ethics Committee of School of Educational Science, Huazhong University of Science and Technology. The studies were conducted in accordance with the local legislation and institutional requirements. The participants provided their written informed consent to participate in this study.

## Author contributions

QT: Writing−original draft, Writing−review and editing. GL: Formal analysis, Writing−review and editing, Conceptualization, Data curation, Methodology. YZ: Conceptualization, Writing−review and editing, Data curation, Formal analysis, Methodology. HS: Writing−review and editing, Conceptualization, Data curation, Formal analysis, Methodology.

## Footnotes

1
https://www.wjx.cn/
