# Supplementary material for: The relation between mental health and career-related stress among prospective graduates in higher education stage during the COVID-19 pandemic: an evidence based on network analysis
Source: Front Psychol. 2024 Jul 8;15:1381846. doi: 10.3389/fpsyg.2024.1381846 (PMC11260747; doi:10.3389/fpsyg.2024.1381846)
Supplement: Supplementary file 1 [file Data_Sheet_1.zip › Supplementary Materials/Supplementary Materials/Table S1.docx]

Table S1. Age distribution of the sample.

| Age Group | n | % |
| --- | --- | --- |
| 18-20 | 119 | 2.94 |
| 21-23 | 3029 | 74.96 |
| 24-26 | 526 | 13.02 |
| 27-52 | 132 | 3.27 |
| Missing | 235 | 5.82 |
| Total | 4041 |  |
