# Supplementary figures and images for: The relation between mental health and career-related stress among prospective graduates in higher education stage during the COVID-19 pandemic: an evidence based on network analysis
Source: Front Psychol. 2024 Jul 8;15:1381846. doi: 10.3389/fpsyg.2024.1381846 (PMC11260747; doi:10.3389/fpsyg.2024.1381846)

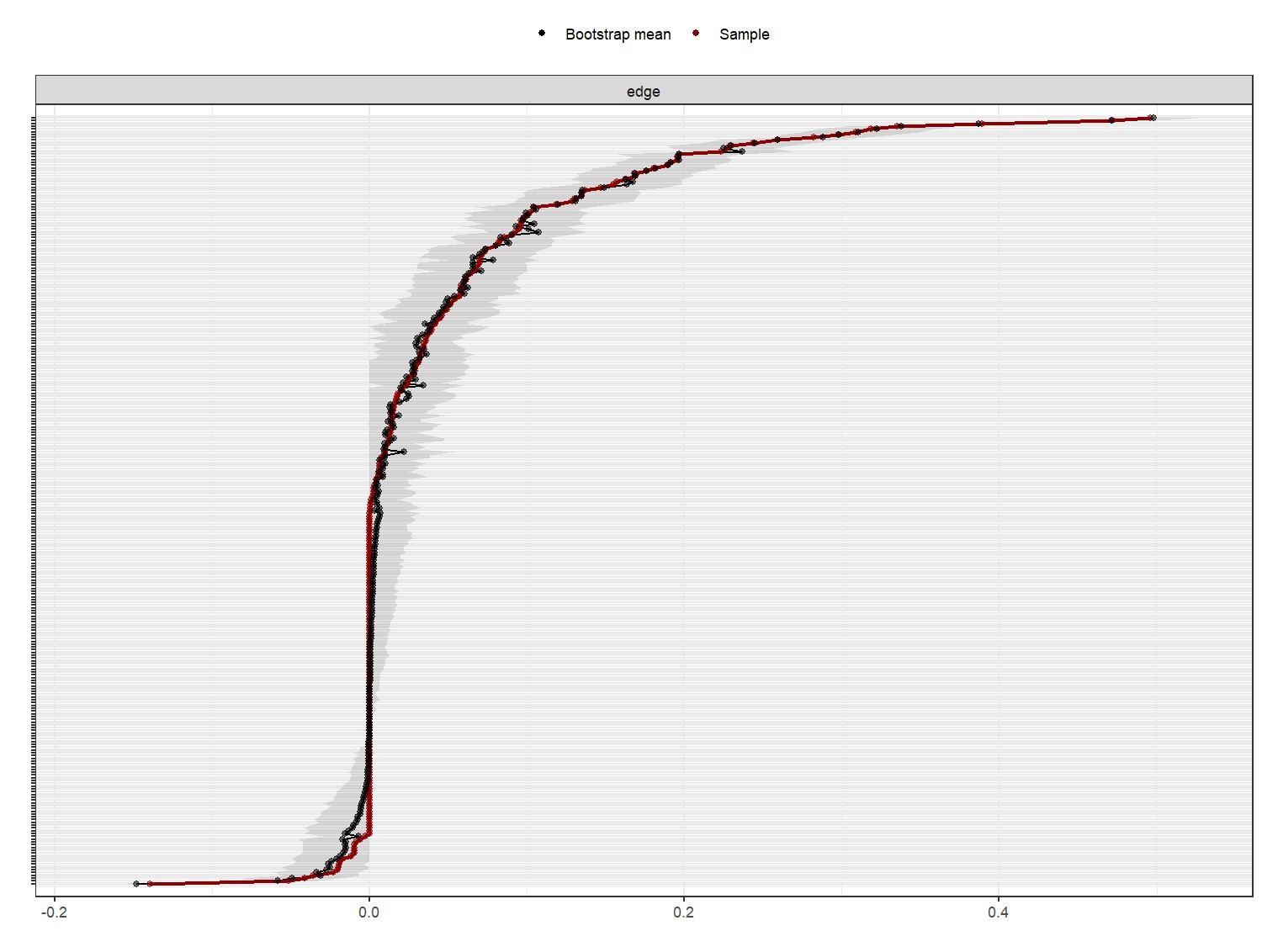

Supplement: Supplementary file 1 [file Data_Sheet_1.zip › Supplementary Materials/Supplementary Materials/Figure S1.jpg]

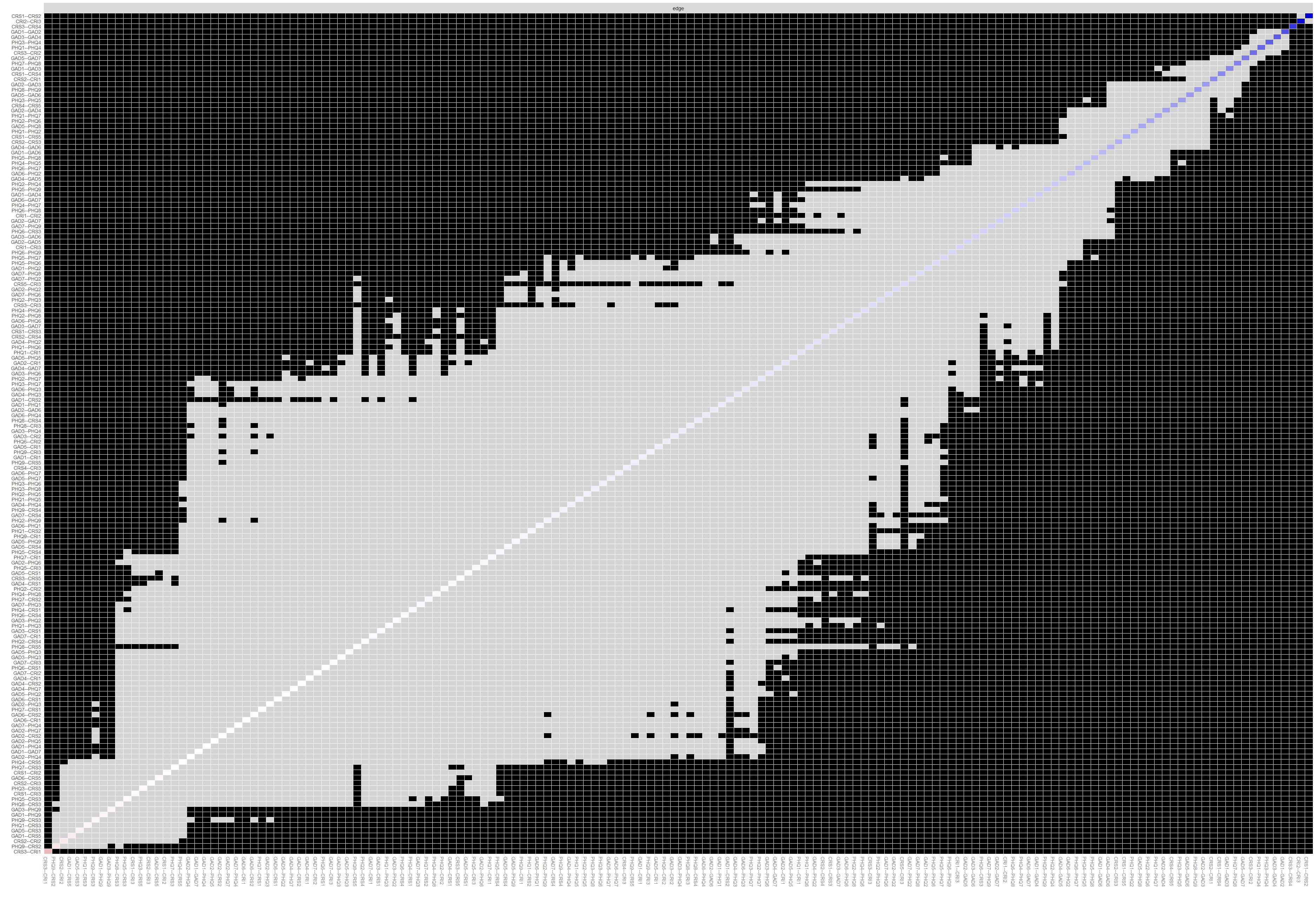

Supplement: Supplementary file 1 [file Data_Sheet_1.zip › Supplementary Materials/Supplementary Materials/Figure S2.jpg]

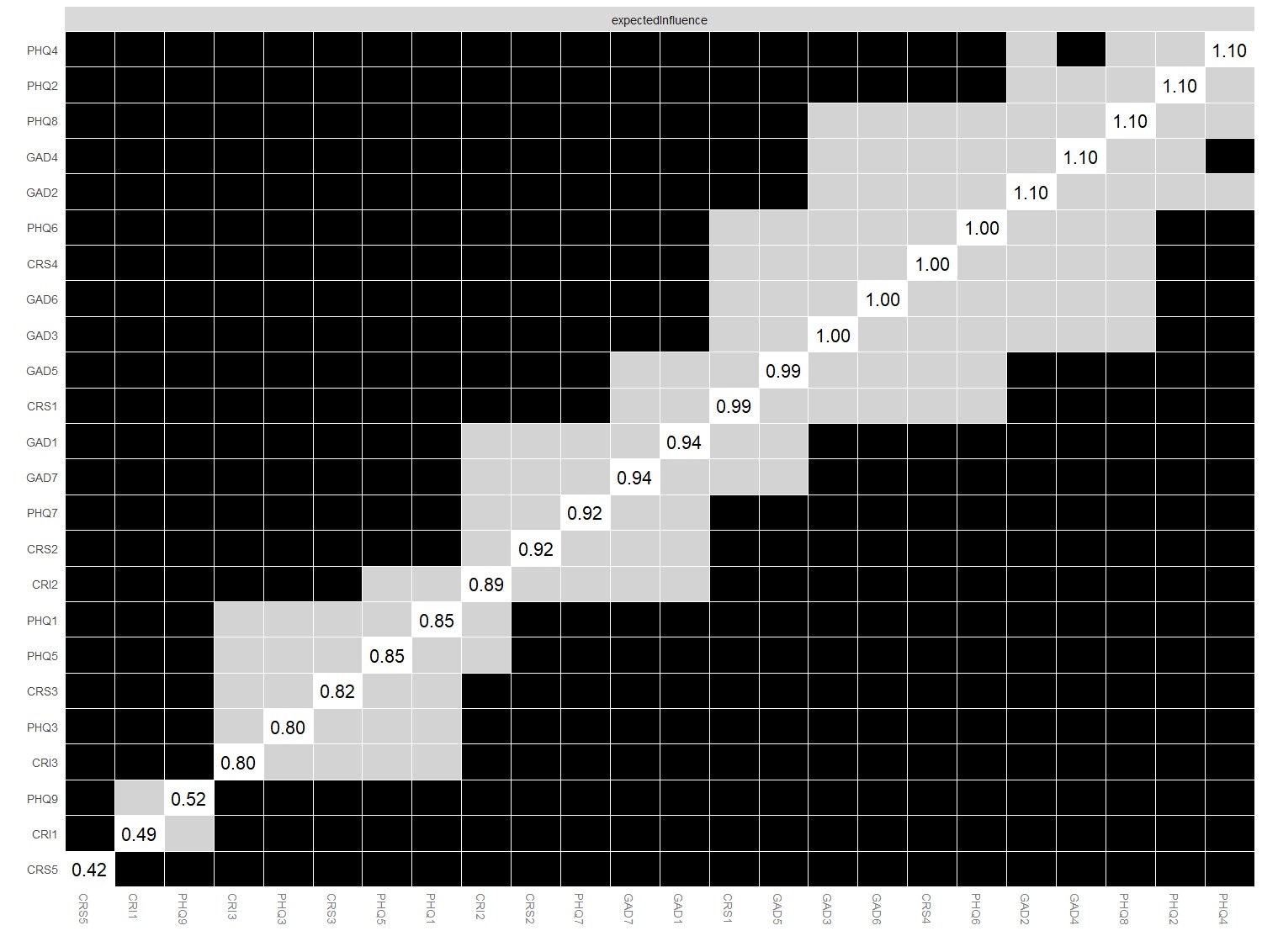

Supplement: Supplementary file 1 [file Data_Sheet_1.zip › Supplementary Materials/Supplementary Materials/Figure S3.jpg]

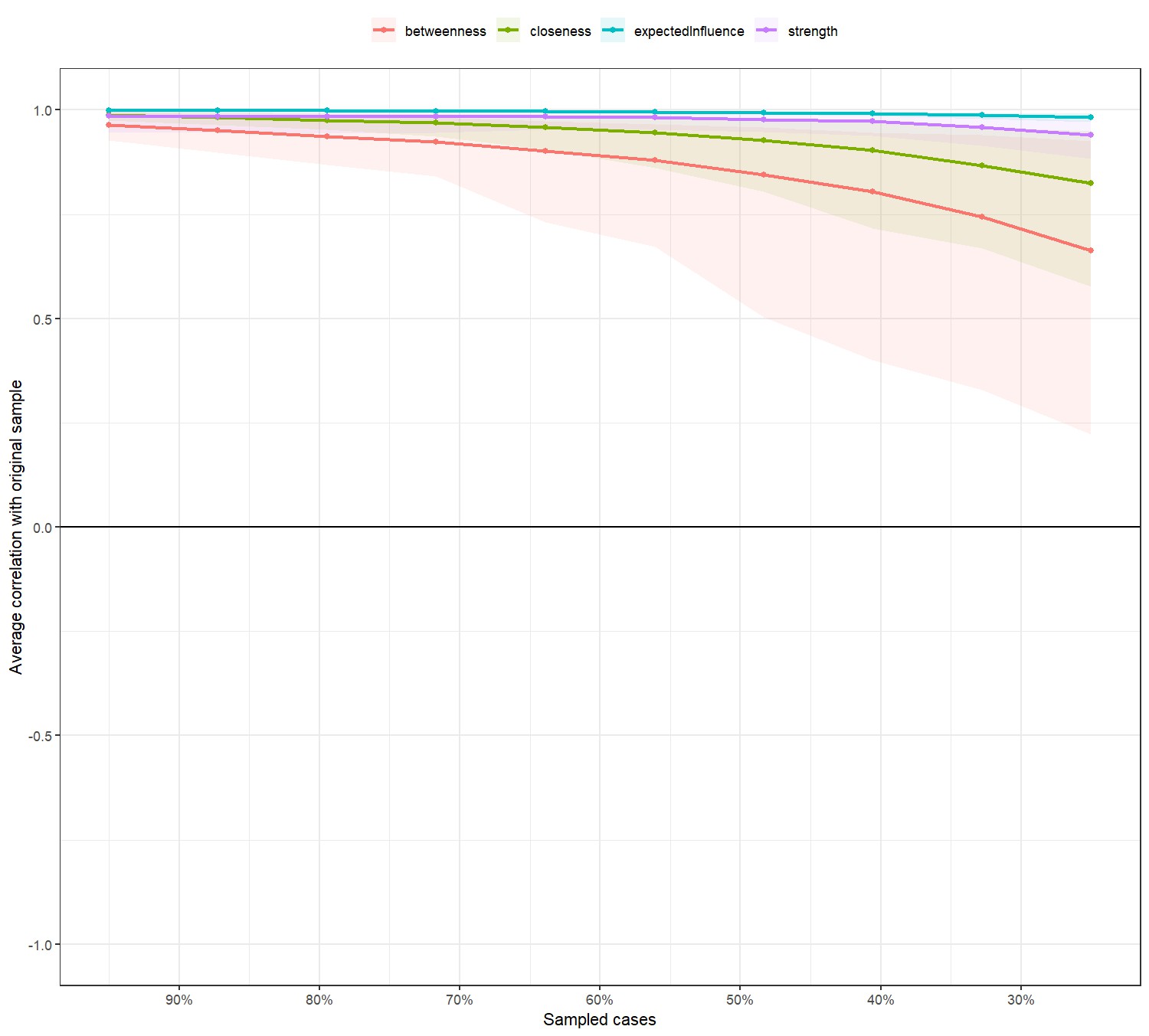

Supplement: Supplementary file 1 [file Data_Sheet_1.zip › Supplementary Materials/Supplementary Materials/Figure S4.jpg]

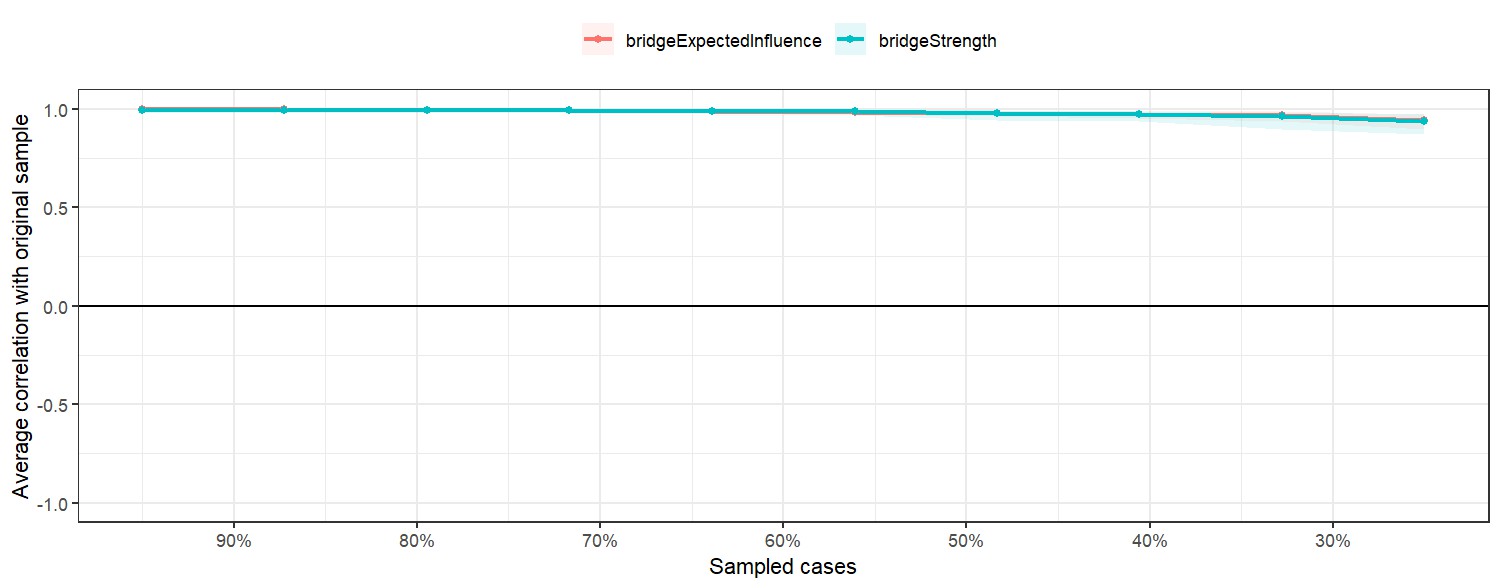

Supplement: Supplementary file 1 [file Data_Sheet_1.zip › Supplementary Materials/Supplementary Materials/Figure S5.jpg]

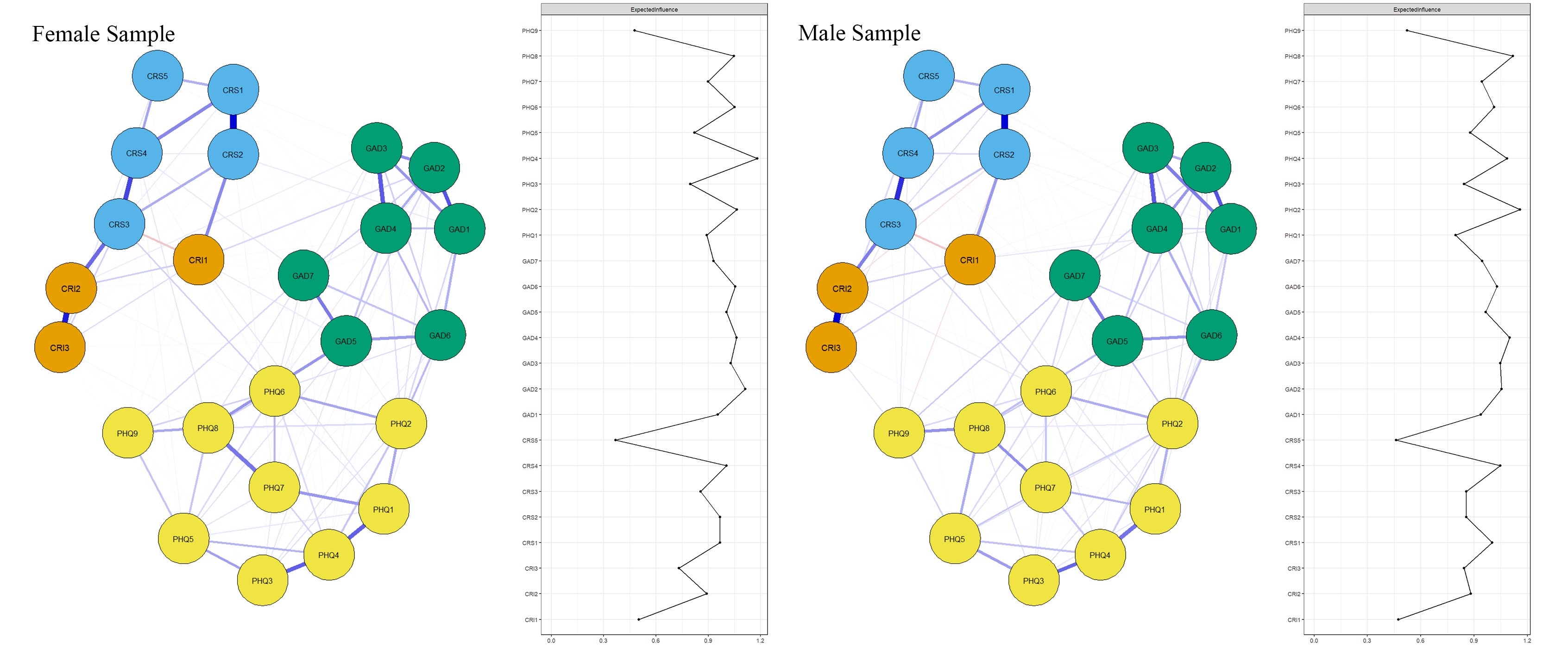

Supplement: Supplementary file 1 [file Data_Sheet_1.zip › Supplementary Materials/Supplementary Materials/Figure S6.jpg]

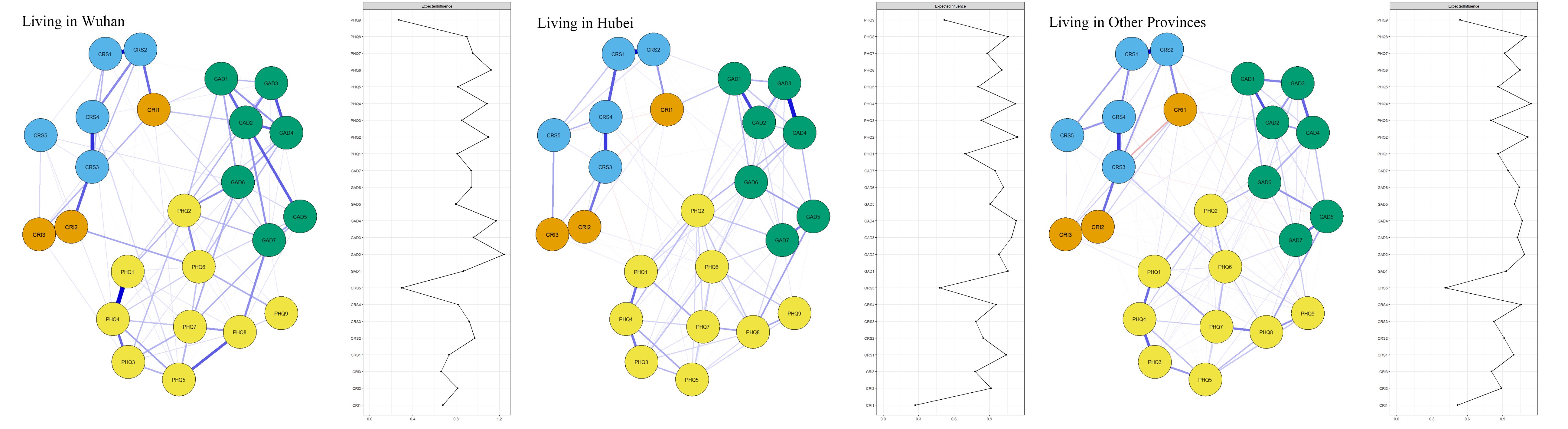

Supplement: Supplementary file 1 [file Data_Sheet_1.zip › Supplementary Materials/Supplementary Materials/Figure S7.jpg]
